# Beyond Antimicrobial Activity: Plant-Derived Bioactive Compounds as Modulators of Antimicrobial Resistance in Broiler Chickens—Mechanisms, Resistome Dynamics, and One Health Implications

**DOI:** 10.3390/plants15213285

**Published:** 2026-10-24

**Authors:** George Cosmin Nadăş, Cosmina Maria Bouari, Nicodim Iosif Fiţ

**Affiliations:** Department of Microbiology, Immunology and Epidemiology, Faculty of Veterinary Medicine, University of Agricultural Sciences and Veterinary Medicine, 400372 Cluj-Napoca, Romania; gnadas@usamvcluj.ro (G.C.N.); C.M.B.; nfit@usamvcluj.ro (N.I.F.)

**Keywords:** plant-derived bioactive compounds, phytochemicals, broiler chickens, antimicrobial resistance, resistome, antimicrobial resistance genes, phytogenic feed additives, One Health

## Abstract

Antimicrobial resistance (AMR) in broiler production is a One Health concern that extends beyond the occurrence of resistant pathogens to the persistence, mobility, and dissemination of antimicrobial resistance genes (ARGs) within intestinal and environmental microbial communities. Plant-derived bioactive compounds are increasingly proposed as alternatives or complements to conventional antimicrobials; however, antibacterial activity against resistant bacteria does not necessarily demonstrate AMR mitigation. This critical narrative review evaluates phytochemicals as resistance-modifying agents in broiler chickens, distinguishing direct antibacterial effects from antibiotic potentiation, resistance-mechanism interference, ARG and mobile genetic element (MGE) modulation, horizontal gene transfer inhibition, and community-level resistome effects. Studies involving selected plant-derived compounds and phytogenic preparations have reported effects on antimicrobial susceptibility, efflux-associated activity, resistance enzymes, plasmid transfer, ARG transcript abundance, and resistome composition across different bacterial species and experimental models. However, these findings are compound-, model-, and endpoint-specific, and direct evidence from broiler chickens remains limited and heterogeneous. Outcomes are strongly dependent on phytochemical composition, dose, formulation, intestinal exposure, bird age, microbial ecology, and analytical methodology, while repeated subinhibitory exposure may under some conditions promote tolerance or antibiotic cross-resistance. From a One Health perspective, reductions in intestinal ARG abundance or resistant bacterial populations should therefore be considered initial rather than definitive evidence of AMR mitigation. Future progress will require chemically standardized interventions evaluated through integrated phenotypic, genomic, transcriptomic, and resistance-mobility approaches. Longitudinal studies across the poultry production continuum are also needed to determine whether these interventions produce durable reductions in resistance burden and dissemination.

## 1. Introduction

Antimicrobial resistance (AMR) has become a major One Health challenge at the interface of animal production, food safety, environmental health, and human medicine, with poultry production systems representing important reservoirs and dissemination pathways for antimicrobial-resistant bacteria and resistance genes [1,2,3]. The extensive use of antimicrobials in food-producing animals creates sustained selective pressure that favors the emergence, persistence, and dissemination of resistant bacterial populations and antimicrobial resistance genes (ARGs), with global antimicrobial use in livestock estimated at approximately 99,500 tonnes in 2020 and projected to exceed 107,000 tonnes by 2030 under current trends [4,5]. Beyond the occurrence of phenotypically resistant bacteria, the broiler intestinal microbiota constitutes a complex reservoir of antimicrobial resistance genes (ARGs), whose persistence and dissemination are shaped by bacterial host composition and by mobile genetic elements (MGEs), including plasmids, integrons, and transposons that facilitate horizontal gene transfer [3,5,6].

In this context, phytogenic feed additives and other plant-derived bioactive compounds have attracted increasing attention as potential alternatives or complements to conventional antimicrobial use in poultry production because of their chemically diverse constituents and their reported antimicrobial, antioxidant, anti-inflammatory, immunomodulatory, and gut microbiota-modulating activities [7,8,9,10]. These preparations contain a wide range of plant secondary metabolites, including polyphenols, flavonoids, phenolic acids, terpenoids, alkaloids, saponins, and tannins. Their biological activity is influenced by botanical source, extraction method, chemical composition, concentration, and formulation [7,11].

However, demonstrating antimicrobial activity against susceptible or even multidrug-resistant bacteria does not necessarily establish that a plant-derived compound mitigates antimicrobial resistance itself. Resistance-modifying activity requires evidence that an intervention alters the resistance phenotype or its underlying determinants—for example, through antibiotic potentiation, efflux-pump inhibition, modulation of resistance-gene expression, or interference with horizontal gene transfer—whereas resistome modulation refers to broader changes in the abundance, diversity, expression, host distribution, or mobility of ARGs within a microbial community [3,10,12,13,14]. This distinction is particularly relevant in broiler production, where phytogenic interventions may reduce pathogen abundance or reshape the intestinal microbiota without necessarily producing measurable changes in the resistome; indeed, recent broiler studies have reported reductions in ARG abundance or expression following some plant-derived interventions, whereas others found little or no persistent effect on resistome composition [12,13,14].

Recent evidence further suggests that some plant-derived compounds may interfere directly with resistance dissemination mechanisms. In poultry-associated experimental systems, trans-cinnamaldehyde, carvacrol, β-resorcylic acid, and caprylic acid reduced conjugative transfer of the *bla*_TEM_ resistance determinant between multidrug-resistant *Salmonella* Heidelberg and *Escherichia coli*, while trans-cinnamaldehyde supplementation also decreased horizontal gene transfer frequency in broiler cecal contents ex vivo, and phytochemical exposure downregulated several conjugation-associated genes [15]. Nevertheless, direct evidence that plant-derived bioactive compounds consistently reduce ARG abundance or mobility, alter resistome composition, or decrease the potential for resistance dissemination in broilers remains limited and heterogeneous, with outcomes varying according to phytochemical composition, experimental model, bird age, microbial community structure, and the analytical approach used to characterize resistance [3,12,13,14,15].

Accordingly, this review critically examines plant-derived bioactive compounds as modulators of antimicrobial resistance in broiler chickens, with particular emphasis on their phytochemical characteristics, resistance-modifying mechanisms, effects on ARGs and MGEs, microbiome–resistome interactions, and potential consequences for resistance dissemination within a One Health framework. Particular attention is given to distinguishing conventional antimicrobial activity from direct evidence of resistance modification and resistome modulation, while identifying methodological limitations and priorities for future research.

## 2. Literature Search Strategy and Evidence Assessment

This manuscript was originally developed as a critical narrative review, with the initial bibliography assembled through purposive selection of publications relevant to plant-derived bioactive compounds, antimicrobial resistance, and poultry production. To improve the transparency and completeness of the evidence base during revision, supplementary literature searches were conducted on 13 October 2026 in PubMed/MEDLINE, Scopus, and Web of Science Core Collection. Nine database-specific searches addressed three complementary domains: (i) plant-derived compounds and antimicrobial resistance-related outcomes in broilers and poultry; (ii) resistance-modifying mechanisms investigated in other bacterial and experimental systems; and (iii) poultry-associated resistome ecology and environmental dissemination within a One Health framework. The search strategies, database fields, publication-date restrictions, and retrieval counts are documented in Supplementary Table S1. In total, 10,326 records were retrieved (PubMed/MEDLINE, *n* = 2094; Scopus, *n* = 4636; and Web of Science Core Collection, *n* = 3596). Automated consolidation using DOIs, PubMed identifiers, database accession numbers, and normalized titles yielded 5564 provisional distinct citation groups, with ambiguous bibliographic matches retained for further verification. Because the database queries did not apply an identical lower publication-date limit, records outside the intended 1990–2026 evidence window were flagged for subsequent eligibility assessment. These retrieval and deduplication counts are reported to improve transparency and should not be interpreted as numbers of studies ultimately included in this critical narrative review.

Priority was given to peer-reviewed original studies examining plant-derived compounds in broilers, poultry-associated bacterial isolates, or poultry-relevant experimental systems. Because direct broiler-specific evidence remains limited for several resistance-modifying mechanisms, mechanistic studies from other bacterial, animal, food, and environmental systems were also considered when they provided biologically relevant evidence on efflux-pump inhibition, antibiotic potentiation, biofilm and quorum-sensing interference, resistance-gene expression, horizontal gene transfer, or resistome modulation. Evidence was interpreted according to its directness, with the highest priority assigned to in vivo broiler studies assessing antimicrobial resistance phenotypes, ARGs, MGEs, or resistome outcomes, followed by poultry-specific ex vivo or isolate-based studies and, finally, mechanistic evidence derived from non-poultry systems. Particular attention was paid to distinguishing conventional antimicrobial activity from direct evidence of resistance modification, ARG/MGE modulation, and community-level resistome effects.

References were selected purposively according to their relevance to the objectives of this critical narrative review. Evidence was prioritized narratively rather than through a formal methodological quality assessment; no standardized risk-of-bias appraisal or study-scoring procedure was performed. Relevant neutral or contrasting findings were also considered to avoid overstating the evidence for antimicrobial resistance mitigation. Articles reporting only general growth-performance, antioxidant, or immunomodulatory effects without a clear link to antimicrobial activity, resistance mechanisms, ARGs, MGEs, or microbial-resistance ecology were not emphasized. When multiple publications addressed similar mechanisms, preference was given to recent studies, primary research, studies providing direct experimental evidence, and investigations using molecular or metagenomic approaches to characterize resistance determinants and, where methodologically supported, identify their microbial hosts.

For interpretative purposes, evidence was grouped into four levels: (i) direct in vivo broiler evidence; (ii) poultry-specific ex vivo or bacterial-isolate evidence; (iii) mechanistic evidence from non-poultry bacterial systems; and (iv) conceptual or extrapolative evidence derived from broader AMR and phytochemical research.

## 3. From Antimicrobial Activity to AMR Mitigation: A Conceptual Framework

The ability of a plant-derived compound to inhibit bacterial growth does not, by itself, demonstrate an effect on antimicrobial resistance. Conventional antimicrobial activity is typically established through endpoints such as growth inhibition, minimum inhibitory concentration (MIC), minimum bactericidal concentration (MBC), or reductions in bacterial burden, whereas resistance-modifying activity requires evidence that a compound alters a resistance phenotype or interferes with a defined resistance mechanism [10,16,17]. Such effects may include restoration of antibiotic susceptibility, reduction in antibiotic MICs when compounds are used in combination, inhibition of multidrug efflux systems or resistance-associated enzymes, modulation of resistance-gene expression, or interference with the acquisition and horizontal transfer of resistance determinants [15,16,17]. At a broader ecological level, AMR mitigation may additionally involve changes in the abundance, expression, host distribution, or mobility of ARGs and MGEs within microbial communities, outcomes that cannot be inferred solely from antibacterial or anti-biofilm activity [3,12,13,14,15].

To avoid conflating these distinct biological outcomes, the evidence considered in this review is organized along a progressive continuum. At the first level, direct antibacterial activity indicates that a phytochemical can inhibit or kill a bacterium, including a resistant isolate, but does not establish reversal or suppression of the resistance mechanism. At the second level, resistance-modifying activity requires demonstration of altered antimicrobial susceptibility or interference with a defined resistance determinant. A third level encompasses effects on resistance dissemination, including changes in ARG expression, plasmid transfer, conjugation, or other MGE-associated processes. Finally, community-level resistome modulation requires evidence of measurable changes in the composition, abundance, expression, host association, or mobility of resistance determinants across the intestinal microbial community [3,12,13,14,15,16]. These levels should not be regarded as interchangeable, because evidence at one level does not necessarily predict an effect at the next.

### 3.1. Antibacterial Activity Against Resistant Bacteria

Plant-derived bioactive compounds have demonstrated antibacterial activity against poultry-associated bacterial isolates described in the original studies as multidrug-resistant (MDR) or extensively drug-resistant (XDR), supporting interest in their potential use as alternatives or adjuncts to conventional antimicrobials [10,17,18,19,20,21,22,23,24]. Throughout this review, MDR and XDR designations are retained as reported by the cited studies and interpreted in relation to the antimicrobial agents and classes included in their susceptibility-testing panels. Because testing panels and classification criteria may differ between studies, these labels should not be assumed to represent universally standardized or directly comparable resistance phenotypes. Such activity is generally demonstrated through growth inhibition, minimum inhibitory concentration (MIC) or minimum bactericidal concentration (MBC) measurements, time–kill assays, reductions in viable bacterial counts, or decreased pathogen burdens in experimentally challenged birds. However, susceptibility of an MDR or XDR isolate to a phytochemical does not itself demonstrate reversal of the underlying resistance phenotype. In such studies, antimicrobial resistance primarily characterizes the bacterial target, whereas the measured outcome reflects direct antibacterial activity of the plant-derived preparation.

Evidence from poultry-associated *E. coli* illustrates both the potential and the variability of these effects. Thyme (*Thymus vulgaris*) and coriander (*Coriandrum sativum*) essential oils were tested against 40 avian *E. coli* isolates, including 24 obtained from broilers, with all broiler isolates classified as MDR; thyme oil, characterized by a high carvacrol content, displayed greater antibacterial activity and lower MIC values than coriander oil [19]. In a broader panel of bacterial pathogens recovered from diseased chickens, cinnamon essential oil showed the lowest median MIC among seven essential oils tested, although marked differences in susceptibility were observed among both bacterial species and individual oils [20]. Garlic (*Allium sativum*) and ginger (*Zingiber officinale*) extracts have also been evaluated against MDR avian pathogenic *E. coli* O78, with garlic showing particularly broad in vitro antibacterial activity and dietary supplementation enhancing bacterial clearance following experimental challenge in broiler chickens [21]. More recently, encapsulated *Lavandula angustifolia* essential oil inhibited clinical *E. coli* isolates displaying varying antimicrobial-resistance profiles, with MIC values of 1–4% (*v*/*v*), while supplementation in broilers was associated with reduced intestinal *E. coli* and coliform counts [24].

Comparable findings have been reported for resistant *Salmonella* and *Campylobacter*. Lemongrass (*Cymbopogon citratus*) essential oil produced substantial reductions in multidrug-resistant *Salmonella enterica* serovar Heidelberg in broth and poultry cecal contents and additionally impaired motility, biofilm formation, and attachment to chicken skin and meat [18]. Oregano essential oil was subsequently evaluated against multidrug-resistant *Salmonella* Pullorum and *Salmonella* Gallinarum in commercial chicken breeders, where both preventive and therapeutic administration reduced the adverse consequences of experimental infection [22]. In broilers challenged with XDR *Campylobacter jejuni*, dietary supplementation with a combination of eugenol and trans-cinnamaldehyde reduced cecal bacterial loads and downregulated several virulence-associated genes [23]. Together, these studies demonstrate that plant-derived preparations can exert biologically relevant antibacterial effects against resistant poultry-associated pathogens across in vitro, ex vivo, and in vivo models [18,19,20,21,22,23,24].

Nevertheless, these findings should not be interpreted as evidence that the corresponding interventions mitigate AMR per se. A reduction in the intestinal population of an MDR pathogen, such as *Salmonella*, demonstrates antibacterial efficacy against that target but does not necessarily indicate a reduction in the overall intestinal resistome. Resistance determinants may persist in other bacterial populations or mobile genetic elements, even when the targeted pathogen declines. Consequently, reduced pathogen counts alone cannot establish decreases in total antimicrobial resistance gene (ARG) abundance, changes in resistome composition, restoration of antibiotic susceptibility, or reduced horizontal gene transfer. Such effects require direct assessment of the corresponding resistance-related endpoints. Moreover, susceptibility to phytochemicals can vary considerably among bacterial species and isolates, and antibiotic resistance does not uniformly predict increased or decreased susceptibility to plant-derived antimicrobials [19,20]. Distinguishing direct antibacterial efficacy from genuine resistance-modifying activity is therefore essential when assessing the contribution of phytogenic interventions to AMR mitigation.

### 3.2. Resistance-Modifying and Antibiotic-Potentiating Activity

Antibiotic-potentiating activity should be distinguished from both intrinsic antibacterial activity and mechanistically demonstrated resistance modification. A reduction in antibiotic minimum inhibitory concentration (MIC), or an interaction identified through checkerboard or time–kill assays, establishes a phenotypic effect under the tested experimental conditions but does not, by itself, demonstrate reversal or inhibition of a specific resistance mechanism. Mechanistically supported resistance modification requires additional evidence, such as direct inhibition of resistance-associated enzymes or target-specific investigation of antimicrobial efflux. Similarly, claims of restored categorical susceptibility require appropriate susceptibility-testing methods and validated interpretative breakpoints. Plant-derived compounds may therefore act as potential antibiotic adjuvants even when their intrinsic antibacterial activity is limited, although phenotypic potentiation should not be equated with confirmed resistance reversal [16,17,25,26,27,28,29,30,31,32].

Evidence from poultry-associated bacteria illustrates the potential of phytochemical–antibiotic combinations, although the methods and endpoints differ across studies. Zych et al. [25] examined interactions between enrofloxacin and cinnamon bark, clove bud, or lavender essential oils against *E. coli* isolates obtained from one-day-old broiler chickens, using checkerboard-derived fractional inhibitory concentration indices (FICIs) and time–kill experiments. Synergy was reported most frequently for lavender oil (82.35% of tested combinations), followed by cinnamon (64.7%) and clove oils (47.1%), while the remaining interactions were classified as additive. Although antibacterial activity was observed at concentrations below the individual MICs, these findings demonstrate in vitro combination effects rather than identifying a specific resistance-modifying mechanism. Kollanoor Johny et al. [26] evaluated the growth of multidrug-resistant *Salmonella* Typhimurium DT104 in broth containing antibiotics at experimentally selected resistance-breakpoint concentrations, together with subinhibitory concentrations of trans-cinnamaldehyde, thymol, carvacrol, eugenol, or β-resorcylic acid. Their growth-based measurements indicated increased antibiotic susceptibility under the experimental conditions but did not constitute a checkerboard-based demonstration of synergy. Palaniappan and Holley [27] used broth microdilution and checkerboard assays to investigate antibiotic susceptibility changes following exposure to selected plant-derived antimicrobials in resistant bacterial strains. Collectively, these studies provide evidence of concentration-dependent antibiotic potentiation, with mechanistic interpretation requiring separate experimental validation.

The mechanisms underlying antibiotic potentiation by plant-derived compounds are not uniform. Altered antimicrobial efflux represents one possible explanation, since active drug extrusion can contribute to multidrug resistance. However, increased intracellular accumulation of ethidium bromide (EtBr) is not specific evidence of efflux-pump inhibition, as dye accumulation may also be influenced by changes in bacterial membrane permeability and integrity. This limitation is particularly relevant for membrane-active compounds such as thymol and carvacrol. Miladi et al. [28] reported concentration-dependent increases in intracellular EtBr accumulation following exposure to thymol and carvacrol, together with two- to eight-fold reductions in tetracycline MICs. These findings are consistent with reduced efflux activity but do not establish direct inhibition of a specific efflux pump. Similarly, in *Salmonella* Typhimurium, carvacrol, thymol, and eugenol enhanced susceptibility to nalidixic acid and increased EtBr accumulation, while also reducing biofilm biomass [32]. The same study reported alterations in bacterial membrane integrity, indicating that membrane-related effects may have contributed to the observed dye accumulation and antibiotic potentiation. Consequently, confirmation of specific efflux-pump inhibition requires complementary experimental approaches, including appropriate membrane-permeability controls and, where feasible, pump-specific functional or genetic assays.

Plant-derived preparations may also potentiate antibiotics by targeting resistance enzymes. Watercress (*Nasturtium officinale*) extracts and 2-phenylethyl isothiocyanate were assessed in combination with antibiotics against extended-spectrum β-lactamase-producing *E. coli* isolates using disk-diffusion and MIC-based antibacterial assays [29]. These results indicate enhanced antibacterial activity in vitro, without establishing inhibition of the underlying β-lactamase mechanism. In contrast, Chandar et al. [30] combined checkerboard assays, used to characterize interactions between plant extracts and meropenem, colistin, or tetracycline, with a separate biochemical assay measuring inhibition of purified NDM-1 activity. Importantly, inhibition of purified NDM-1 provides direct biochemical evidence of enzyme inhibition, whereas synergy between plant extracts and meropenem against an NDM-producing bacterial strain demonstrates phenotypic antibiotic potentiation but does not, by itself, establish NDM inhibition. More recently, Wei et al. [31] investigated primin and meropenem using MIC determination, checkerboard and time–kill assays, together with NDM-5 enzyme-inhibition experiments, binding analyses, and site-directed mutagenesis. The authors also evaluated treatment efficacy in a murine infection model. Thus, the evidence supporting NDM-5 inhibition derives from complementary biochemical and molecular experiments rather than from the observed synergy alone; these findings nevertheless remain distinct from direct evidence of antimicrobial resistance mitigation in broiler chickens.

The interpretation of phytochemical–antibiotic interactions requires explicit distinction between synergy, additivity, indifference, and antagonism. Synergy refers to an interaction exceeding the predefined expectation for combined activity, whereas additivity indicates an effect consistent with the contributions of the individual agents. Indifference denotes the absence of a meaningful interaction according to the criteria applied, while antagonism indicates a reduction in antibacterial effectiveness associated with the combination. In checkerboard assays, these categories are generally assigned using fractional inhibitory concentration indices (FICIs), although the numerical thresholds may vary between studies and should therefore be interpreted according to the definitions used by the original authors. Time–kill assays assess changes in viable bacterial counts over time and use different criteria; their results should not be considered directly interchangeable with checkerboard classifications. Moreover, inhibition at concentrations below the individual MICs does not independently establish synergy, and neither checkerboard nor time–kill synergy identifies the underlying molecular mechanism. Confirmation of resistance modification requires complementary mechanistic experiments, such as enzyme-inhibition assays, appropriately controlled efflux studies, or genetic validation. Because much of the available evidence derives from in vitro or non-poultry experimental models, antibiotic potentiation should not be interpreted as demonstrating sustained resistance reversal or resistome-level AMR mitigation in broiler chickens [25,26,27,28,29,30,31,32].

### 3.3. Antimicrobial Resistance Genes and Horizontal Gene Transfer

At the genetic level, mitigation of antimicrobial resistance requires more than suppression of resistant bacterial growth or restoration of antibiotic susceptibility. Antimicrobial resistance genes (ARGs) encode specific resistance functions, whereas their persistence and dissemination within microbial communities are strongly influenced by mobile genetic elements (MGEs), including plasmids, transposons, insertion sequences, and integron-associated gene cassettes capable of facilitating horizontal gene transfer between bacterial hosts [3,5,6]. Consequently, evaluation of plant-derived interventions should extend beyond changes in bacterial abundance to include ARG abundance and expression, ARG–host associations, linkage of resistance determinants to MGEs, plasmid prevalence and stability, and the frequency of horizontal transfer [12,13,14,15,33,34,35,36,37,38]. These endpoints provide increasingly direct evidence that a phytogenic intervention affects the maintenance or dissemination of resistance determinants rather than merely reducing the population size of bacteria that carry them.

Broiler studies provide emerging but still heterogeneous evidence that phytogenic interventions can influence ARGs and plasmid-associated resistance. In an in vivo study using commercial phytogenic feed additives containing carvacrol, cinnamaldehyde, and eugenol, Saliu et al. [33] observed a lower prevalence of extended-spectrum β-lactamase-producing Enterobacteriaceae in supplemented broilers, although effects on ex vivo plasmid conjugation were less consistent and combinations of phytogenic additives with direct-fed microbials did not uniformly decrease transfer frequency. This distinction is important because a reduction in the prevalence of bacteria harboring resistance plasmids does not necessarily imply inhibition of plasmid transfer itself. At the community level, shotgun metatranscriptomics showed that phytogenic supplementation reduced the abundance of ARG transcript abundance in the broiler gut compared with control and bacitracin-treated groups [12], while *Neolamarckia cadamba* leaf extract altered selected ARGs, MGEs, and their associations with potential bacterial hosts in cecal contents and feces [13]. More recently, supplementation with *Pulsatilla* saponins combined with a herbal formulation reduced the abundance and diversity of multidrug-resistance-related ARGs relative to antibiotic supplementation and was associated with restructuring of the cecal microbial community [34].

A particularly relevant target is plasmid-mediated conjugation, because inhibition of this process may reduce resistance dissemination even at concentrations that do not inhibit bacterial growth. In poultry-associated experimental systems, trans-cinnamaldehyde, carvacrol, β-resorcylic acid, and caprylic acid reduced transfer of a *bla*_TEM-1B_-carrying plasmid between multidrug-resistant *Salmonella* Heidelberg and *E. coli* in broth, chicken manure, and water, while in-feed trans-cinnamaldehyde supplementation also reduced transfer frequency in broiler cecal contents ex vivo [15]. Phytochemical exposure also downregulated conjugation-associated genes and reduced pilus formation [15]. However, reduced plasmid transfer at subinhibitory concentrations does not necessarily demonstrate specific inhibition of the conjugation machinery, as indirect effects on bacterial physiology, membrane integrity, metabolism, or cell-to-cell interactions may contribute. Interpretation therefore requires appropriate controls for donor and recipient growth and viability, donor-to-recipient ratios, plasmid stability, bacterial aggregation, and membrane integrity. As mechanistic evidence outside the poultry model, Skalicka-Woźniak et al. [35] reported that commercially sourced linalool, also identified as a constituent of *Thymus vulgaris* essential oil, reduced pKM101 plasmid transfer between *E. coli* strains by approximately 84% in vitro. Similarly, nanostructured lipid carriers containing sage or olibanum essential oils markedly reduced conjugation of *bla*_TEM_-associated resistance in experimental food matrices, including systems containing chicken-derived material [36].

Recent mechanistic studies further strengthen the biological plausibility of phytochemical-mediated inhibition of ARG mobility. Carvacrol and thymol, applied at subinhibitory concentrations, reduced intra- and inter-generic transfer of *bla*_NDM_-positive IncX3 plasmids without suppressing bacterial growth; the effect was associated with reduced ATP availability, altered membrane permeability, and downregulation of pathways related to reactive oxygen species generation and the SOS response, and inhibition by carvacrol was also demonstrated in an animal gastrointestinal model [37]. In parallel, screening of plant-derived flavonoids identified broad anti-conjugation activity, with hydroxylated flavonoids showing stronger effects than more lipophilic analogues. Scutellarein was shown to inhibit plasmid transfer through disruption of iron homeostasis, induction of zinc-responsive envelope stress, altered redox metabolism, and reduced bacterial adhesion and aggregation; inhibition was confirmed both in vitro and in murine intestinal models [38]. These studies indicate that phytochemicals may influence ARG dissemination through multiple mechanisms acting on bacterial energetics, membrane physiology, stress responses, cell-to-cell contact, and conjugation-associated gene expression [35,36,37,38]. These findings should be interpreted according to the experimental model. In vitro conjugation assays using defined donor–recipient systems [15,35] should be distinguished from poultry-associated experiments in manure, water, or chicken-derived food matrices [15,36]. In study [15], broilers received dietary trans-cinnamaldehyde, but plasmid transfer was subsequently assessed in cecal contents ex vivo; therefore, these results do not establish inhibition of horizontal gene transfer within the intact broiler intestine in vivo. Findings from non-poultry animal models [37,38] provide proof-of-principle mechanistic evidence rather than evidence of efficacy in broiler production, and require further validation in poultry.

Nevertheless, interpretation of these findings requires caution. A decrease in the relative abundance of an ARG may result from depletion of its bacterial host rather than elimination of the gene, whereas detection of an ARG does not establish transcriptional activity, genomic location, or capacity for horizontal transfer. Likewise, the presence of plasmids, integrons, or other MGEs indicates mobility potential but does not demonstrate that transfer is actively occurring. Conversely, conjugation assays usually evaluate defined donor–recipient–plasmid combinations and may not reproduce the complexity of the intestinal microbial community. Future broiler studies should therefore integrate absolute ARG quantification, shotgun metagenomics, metatranscriptomics, plasmidome or long-read sequencing, ARG–host linkage, and functional conjugation assays to determine whether phytogenic interventions genuinely reduce the persistence and transmissibility of resistance determinants [3,6,12,13,14,15,33,34,35,36,37,38].

### 3.4. Community-Level Resistome Effects

Community-level resistome analysis provides a broader assessment of antimicrobial resistance than measurements of individual ARGs because it evaluates the composition, diversity, abundance, expression, and ecological distribution of resistance determinants across the intestinal microbial community. Such effects may result from direct modulation of resistance determinants, indirect restructuring of ARG-carrying bacterial populations, or both; consequently, microbiome changes and resistome changes should be considered related but distinct outcomes [3,6,12,13,14].

Available broiler studies indicate that phytogenic interventions can modify resistome structure, although responses are heterogeneous. Phenolic-rich blueberry and blackberry pomace extracts produced cecal resistome profiles that differed from those observed in birds receiving antibiotic growth promoters, with the latter showing greater enrichment of several resistance categories [39]. Subsequent studies using cranberry and blueberry pomaces likewise reported treatment-associated changes in ARG abundance and diversity, but effects were gene specific rather than uniformly suppressive, with some resistance determinants decreasing while others increased [40,41]. Metatranscriptomic analysis further showed that a commercial phytogenic feed additive reduced ARG transcript abundance relative to control and bacitracin-treated groups [12], while *Pulsatilla* saponins combined with herbal supplementation were associated with reduced abundance and diversity of multidrug-resistance-related ARGs [34]. In contrast, a recent shotgun-metagenomic study detecting 823 ARGs found no significant effect of an essential-oil blend on resistome α- or β-diversity, whereas bird age exerted a substantially stronger influence on resistome structure [14].

These contrasting findings indicate that phytogenic supplementation should not be assumed to produce a consistent reduction in the broiler resistome. Resistome composition is influenced by multiple ecological and methodological factors, including diet, antimicrobial exposure, farm environment, litter, housing conditions, breed, bird age, intestinal sampling site, sequencing depth, and bioinformatic approaches. Consequently, changes in relative ARG abundance may reflect shifts in ARG-carrying bacterial populations or methodological variability rather than elimination of resistance determinants [14,42]. Conventional short-read metagenomics can characterize ARG profiles but often cannot reliably assign resistance determinants to specific bacterial hosts, plasmids, or other mobile genetic elements. Future studies should therefore combine longitudinal resistome profiling and absolute ARG quantification with complementary approaches, such as long-read sequencing or isolate-based validation, to establish ARG–host–MGE associations more reliably [6,43]. The resulting evidence continuum, from direct antibacterial activity through resistance modification and ARG/MGE modulation to community-level resistome effects, is summarized in Figure 1.

## 4. Phytochemical Determinants of Resistance-Modifying Activity

### 4.1. Major Phytochemical Classes and Structure-Activity Relationships

The resistance-modifying potential of plant-derived bioactive compounds cannot be predicted solely from botanical origin or broad phytochemical class. Instead, it depends on molecular characteristics, substitution pattern, lipophilicity, ionization state, and polar surface area, as well as the relative abundance of individual constituents within complex extracts [11,16,17,44,45,46,47,48,49]. Compounds associated with antibacterial or resistance-modifying effects encompass chemically diverse groups, including phenolic monoterpenes, polyphenols and flavonoids, alkaloids, tannins, isothiocyanates, quinones, and saponins. Importantly, compounds within the same phytochemical class may differ substantially in intrinsic antibacterial potency and in their ability to potentiate antibiotics or interfere with resistance mechanisms. Phytochemical class should therefore be regarded as a useful organizational framework rather than as a predictor of AMR-modifying activity.

Among terpenoid-derived compounds, the phenolic monoterpenes carvacrol and thymol illustrate how relatively subtle structural characteristics can influence biological activity. Both isomers possess a hydrophobic aromatic scaffold and a phenolic hydroxyl group and have been associated with membrane perturbation, antibiotic potentiation, and changes in EtBr accumulation consistent with altered efflux-related activity; however, these effects cannot be attributed specifically to efflux-pump inhibition without appropriate controls for membrane permeability [28,37,44]. Polyphenols display an even broader range of structural determinants. For flavonoids, hydroxylation at C5, C7, C3′, and C4′ and prenylation or geranylation at specific positions have been associated with increased antibacterial activity, whereas some methoxylation patterns can decrease activity [45]. Structure–activity relationships are mechanism- and endpoint-specific; structural features associated with direct antibacterial activity cannot automatically be considered predictive of antibiotic potentiation, efflux modulation, β-lactamase inhibition, or inhibition of plasmid conjugation. In a study of 37 prenylated (iso)flavonoids, Irianti et al. [46] developed binary QSAR models for ciprofloxacin potentiation and ethidium bromide accumulation as potential NorA-associated endpoints. Both models achieved 78% accuracy under leave-one-out cross-validation. However, no independent external validation was performed, and the authors acknowledged that validation using additional compounds is required. Accordingly, the identified molecular descriptors should be regarded as associations within the investigated dataset rather than universally established determinants of NorA inhibition.

Alkaloids further illustrate the importance of chemical context and interactions among plant constituents. Berberine is a cationic isoquinoline alkaloid with antibacterial activity but is also readily extruded by bacterial multidrug efflux systems. In *Berberis* spp., the co-occurring flavonolignan 5′-methoxyhydnocarpin inhibited the NorA efflux pump and markedly increased intracellular accumulation and antibacterial activity of berberine, demonstrating that resistance-modifying activity can arise from complementary constituents within the same botanical source [47]. Among polyphenols, epigallocatechin gallate enhanced β-lactam activity against methicillin-resistant *S. aureus* through effects on cell-wall integrity and peptidoglycan rather than suppression of *mecA*-encoded PBP2a expression [48]. Other chemically distinct molecules, including 2-phenylethyl isothiocyanate [29], the benzoquinone primin [31], and plant-derived saponins [34], further expand the chemical space associated with AMR-relevant effects. Overall, available evidence indicates that resistance modification is determined more reliably by defined chemical composition and molecular properties than by the designation of a preparation as an essential oil, polyphenol-rich extract, or phytogenic additive [16,45,46,49].

### 4.2. Extraction, Standardization, Formulation, Stability, and Intestinal Exposure

The biological activity of a plant-derived preparation depends not only on the phytochemical structures discussed above but also on how those compounds are obtained, characterized, and formulated. Extraction solvent, temperature, extraction time, particle size, and conventional versus assisted extraction procedures can substantially alter the qualitative and quantitative phytochemical profile of an extract and, consequently, its antimicrobial activity [11,50]. This variability is particularly important for essential oils and complex botanical preparations, whose composition is additionally influenced by genotype or chemotype, geographical origin, environmental conditions, phenological stage, harvest time, storage, and distillation procedures [51,52]. For example, essential oils obtained from different *Thymus* species and geographical locations showed marked differences in the proportions of thymol, carvacrol, *p*-cymene, and linalool, accompanied by differences in antimicrobial potency [52]. Thus, botanical species names alone are insufficient to define an experimentally reproducible AMR-modifying intervention. Batch-to-batch variability should therefore be documented through chemical profiling and quantification of major constituents. Moreover, findings obtained with purified individual compounds should not be considered directly equivalent to those obtained with complex botanical preparations, whose biological activity may depend on constituent ratios and interactions.

Formulation introduces an additional source of variability because many bioactive constituents, particularly essential-oil components, are volatile, hydrophobic, poorly water-soluble, and susceptible to degradation during processing, storage, and passage through the gastrointestinal tract [51]. Encapsulation, liposomal delivery, nanoemulsions, and related carrier systems have therefore been developed to increase stability, improve dispersibility and bioavailability, and permit more controlled release within the gastrointestinal tract [53,54,55]. Low systemic bioavailability is not necessarily a limitation for gut-directed interventions, provided that effective concentrations are achieved and maintained at the relevant intestinal site. Therefore, local intestinal exposure, rather than systemic bioavailability alone, should be considered when evaluating phytochemical effects on intestinal antimicrobial resistance. In broilers, matrix-encapsulated phytogenic additives containing carvacrol, thymol, and limonene produced different biological responses from a powdered formulation containing menthol and anethole [53], while liposomal encapsulation of oregano, cinnamon, and clove essential oils altered intestinal bacterial populations and improved several gut-associated outcomes [54]. Encapsulated *Lavandula angustifolia* essential oil has likewise been evaluated directly in broilers [24]. These studies demonstrate that formulation can materially influence exposure and biological response, although improved delivery or gut-health effects should not themselves be interpreted as evidence of AMR mitigation.

Accordingly, reproducibility in phytochemical AMR research requires substantially greater chemical and formulation standardization than is provided by generic descriptors such as “plant extract”, “essential oil”, or “phytogenic feed additive”. Studies should report botanical identity and plant part, extraction procedure, analytical characterization of major constituents, concentration and administered dose, formulation or carrier system, and, where possible, stability and gastrointestinal release characteristics [11,50,51,55]. For resistance-modifying effects, particularly inhibition of plasmid conjugation, effective concentrations demonstrated in vitro should be distinguished from concentrations incorporated into feed, estimated intestinal exposure, and analytically measured intestinal concentrations. Feed inclusion levels do not establish whether active compounds reach the relevant intestinal compartment at effective concentrations, for a sufficient duration, and in an appropriate chemical form. Where intestinal exposure data are unavailable, extrapolation of in vitro anti-conjugation effects to broiler conditions remains uncertain. Failure to characterize these variables may partly explain apparently contradictory results among phytogenic interventions and limits meaningful comparison across antimicrobial, ARG, and resistome studies. The principal phytochemical classes, representative compounds, physicochemical characteristics, and AMR-relevant activities discussed in this review are summarized in Table 1.

## 5. Bacterial Adaptation to Plant-Derived Antimicrobials: Tolerance and Cross-Resistance

Although the multi-target activity of many plant-derived antimicrobials is often considered less conducive to resistance development than the highly specific modes of action of conventional antibiotics, bacterial adaptation to phytochemicals cannot be excluded [56]. This issue is particularly relevant when microorganisms are exposed repeatedly to subinhibitory concentrations, which may arise from insufficient dosing, compound degradation, interactions with feed or intestinal contents, rapid absorption, or incomplete release from delivery systems [51,55,56]. Such exposure may produce transient physiological adaptation or tolerance without a substantial increase in MIC, whereas prolonged selection can, under some conditions, generate stable heritable resistance. Cross-resistance is of greater AMR concern because adaptation to a phytochemical may simultaneously alter susceptibility to antibiotics or unrelated environmental stresses [56].

Experimental studies demonstrate that these outcomes are highly compound- and strain-dependent. Growth of *Salmonella enterica* serovar Typhimurium in sublethal thymol, carvacrol, citral, or eugenol induced remodeling of membrane fatty-acid composition and increased tolerance to the biocides peracetic acid and didecyl dimethyl ammonium bromide [57]. Similarly, *E. coli* O157:H7 adapted to sublethal thymol, carvacrol, or trans-cinnamaldehyde developed increased resistance to subsequent lethal exposure to these compounds and cross-protection against heat and oxidative stress, accompanied by extensive transcriptional changes involving membrane, oxidative-stress, heat-shock, and iron-metabolism pathways [58]. However, adaptation is not an inevitable consequence of phytochemical exposure. *S.* Typhimurium exposed to subinhibitory oregano essential oil or carvacrol showed little evidence of acquired direct tolerance or cross-protection against heat, acid, or osmotic stress [59]. Likewise, although adaptation of *E. coli* O157:H7 to thymol, carvacrol, and trans-cinnamaldehyde altered expression of stress- and virulence-associated genes, subsequent phenotypic testing did not demonstrate increased antibiotic resistance and showed reduced efflux-pump activity and biofilm formation [60].More concerning from an AMR perspective, cyclic exposure of *S.* Typhimurium to carvacrol selected stable resistant variants; one evolved lineage also showed reduced susceptibility to multiple antibiotic classes, while whole-genome sequencing identified mutations affecting stress-response regulation [61]. Subsequent adaptive-evolution experiments showed that carvacrol-resistant *S.* Typhimurium variants can arise through different mutational trajectories, with *lon* and *rob* mutations experimentally linked to increased resistance to carvacrol and reduced susceptibility to several antibiotics [62]. However, increased antibiotic MICs in laboratory-evolved lineages do not necessarily indicate clinically relevant resistance. Clinical resistance must be interpreted using validated organism–antibiotic-specific clinical breakpoints, whereas epidemiological cutoff values distinguish wild-type from non-wild-type susceptibility without necessarily predicting clinical treatment failure.

Taken together, available evidence supports neither the assumption that resistance to plant-derived antimicrobials is unlikely to evolve nor the opposite assumption that phytochemical use inevitably selects antibiotic resistance [56,57,58,59,60,61,62]. The outcome appears to depend on compound structure, concentration, duration and pattern of exposure, bacterial genotype, and the environmental matrix. Importantly, direct evidence of such adaptive evolution under broiler intestinal conditions remains scarce. Future poultry studies should therefore incorporate repeated sub-MIC exposure experiments, longitudinal susceptibility testing, whole-genome sequencing of evolved isolates, and assessment of antibiotic cross-resistance before long-term phytogenic interventions are considered intrinsically low-risk from an AMR perspective.

These contrasting exposure-dependent outcomes are summarized in Figure 2, illustrating how dose and exposure duration, formulation and bioavailability, bacterial genotype, and the intestinal environment may shift phytochemical exposure toward either antimicrobial efficacy or adaptive responses associated with tolerance and potential cross-resistance.

## 6. One Health Implications: From the Broiler Gut to the Environment and Food Chain

The relevance of phytochemical-mediated AMR modulation in broilers ultimately depends on whether intestinal effects translate into reduced dissemination of resistant bacteria and resistance determinants beyond the bird. The broiler production continuum connects the intestinal resistome with feces, litter, farm dust, surrounding soil, processing environments, manure-amended agricultural land, water, and potentially plant-associated microbiomes [2,3,63,64,65]. Shotgun metagenomic analysis across 26 commercial poultry farms and a processing plant identified thousands of ARGs throughout vertically integrated production systems, although evidence of direct farm-to-fork transmission was limited and effective biosecurity appeared to maintain separation between internal and external farm environments in most cases [63]. Airborne dissemination also deserves consideration: farm-dust metagenomics from European broiler farms demonstrated a diverse resistome partly related to fecal sources and on-farm antimicrobial use, indicating that AMR exposure pathways are not restricted to litter or manure [65].

Land application of poultry litter represents a particularly important environmental interface. Broiler litter can retain resistant bacteria, ARGs, and MGEs during storage, although its microbiome and resistome change substantially with stockpile age and spatial conditions [64]. Field and experimental studies have reported ARG enrichment and overlapping ARG detection in poultry litter, agricultural soils, and plant-associated compartments. These findings are consistent with possible dissemination but do not independently establish horizontal gene transfer or directional transmission between compartments. Tripathi et al. [66], using sequencing and digital PCR, tracked several ARGs across poultry litter, soil, and sorghum tissues, while a recent shotgun-metagenomic field study showed that poultry-litter amendment altered the soil microbiome and enriched multiple resistance categories [67,68]. However, environmental dissemination is not uniform: a metagenomic investigation of poultry production sites in the southeastern United States found no detectable persistence of litter-associated microorganisms or ARGs in adjacent soils and downstream waters several months after litter application [69]. These contrasting findings emphasize that environmental fate depends on manure treatment, soil properties, microbial ecology, application practices, and time after exposure.

The soil–plant interface may provide a further route toward the food chain. Poultry-manure amendment has been associated with increased ARG abundance in rhizosphere, root endophyte, and phyllosphere communities of lettuce [70], while field studies using chicken litter have detected persistent resistance genes in soil and, to a more limited extent, on harvested vegetables [67]. More recent evidence indicates enrichment of tetracycline-, macrolide-, and multidrug-resistance determinants across poultry-litter-amended soil and maize compartments [71]. Detection of shared ARGs across compartments indicates overlapping resistance profiles and may suggest ecological associations. Such findings may be consistent with dissemination, but demonstrated transmission requires additional evidence establishing source linkage and directionality. Detection of the same ARG in two compartments does not, by itself, demonstrate horizontal gene transfer or farm-to-fork transmission. These studies provide valuable contextual evidence but do not establish clinically meaningful human exposure or farm-to-fork transmission. Likewise, reduced intestinal ARG abundance in broilers does not, by itself, demonstrate reduced dissemination to the environment or transmission to humans. Demonstrating genuine AMR mitigation will require longitudinal tracing from the broiler gut through feces and litter to environmental compartments, together with assessment of ARG persistence, host association, mobility, and potential entry into the food chain [63,64,65,66,67,68,69,70,71]. The principal pathways linking the broiler gut resistome with environmental compartments, the food chain, and potential human or animal exposure are summarized in Figure 3, together with the current strength of evidence across this One Health continuum.

## 7. Broiler-Specific Evidence for AMR Modulation

When the evidence base is restricted to broiler chickens, the number of studies directly demonstrating AMR modification is considerably smaller than the broader literature describing antimicrobial or microbiota-modulating effects of phytogenic interventions. One of the most informative in vivo studies evaluated encapsulated cinnamaldehyde and citral in broilers and combined phenotypic susceptibility testing with whole-genome sequencing of *E. coli* isolates [72]. Cinnamaldehyde alone or in combination with citral was associated with lower frequencies of resistance to several β-lactams, gentamicin, and sulfonamides than the control or bacitracin treatments, while some ARGs and plasmids were also less prevalent in recovered isolates [72]. However, resistance prevalence declined with bird age across treatments, emphasizing the need to distinguish dietary effects from normal temporal succession. In another large challenge experiment, an organic-acid feed additive containing cinnamaldehyde did not increase the abundance of antibiotic-resistant *E. coli* and was associated with lower ceftriaxone MICs than antibiotic treatment, although the mixed formulation prevents attribution of the response specifically to cinnamaldehyde [73]. Similarly, phytogenic products containing carvacrol, cinnamaldehyde, and eugenol reduced the prevalence of ESBL-producing Enterobacteriaceae in broilers, but their influence on ex vivo plasmid conjugation was inconsistent [33]. Together, these studies provide direct evidence that phytogenic interventions can influence resistant bacterial populations and, in some cases, resistance genotype or plasmid carriage, but they do not establish uniform reversal of resistance across antimicrobial classes.

Recent studies examining antimicrobial resistance genes (ARGs) and the broiler gut resistome have reported variable responses to phytogenic interventions. Metatranscriptomic analysis demonstrated changes in the broiler gut resistome at the transcript level following phytogenic supplementation [12], while *Neolamarckia cadamba* leaf extract altered selected ARGs, MGEs, and their associations with microbial taxa in cecal contents and feces [13]. Dietary *Pulsatilla* saponins combined with herbal supplementation were likewise associated with reductions in the abundance and diversity of several multidrug-resistance-related ARGs [34]. Berry-derived polyphenol-rich interventions have produced measurable changes in cecal resistome profiles [39,40,41], whereas a recent shotgun-metagenomic study found no persistent effect of an essential-oil blend on overall resistome α- or β-diversity and identified bird age as a stronger determinant of resistome structure [14]. At the level of resistance mobility, plant-derived compounds have reduced conjugative transfer in poultry-associated matrices and broiler cecal contents ex vivo [15], but demonstration of sustained inhibition of HGT within the intact broiler intestinal ecosystem remains lacking. Thus, the strongest community-level evidence currently supports the conclusion that phytogenic interventions can alter selected components of the broiler resistome, but not that they consistently reduce total resistance burden or dissemination potential.

A larger body of poultry-specific evidence concerns activity against resistant bacterial targets rather than modification of resistance itself. Allicin and cinnamon oil inhibited MDR *E. coli* isolated from diseased broilers and reduced expression of the integron-associated *intI1* gene together with the virulence-associated *fimH* and *luxS* genes in vitro [74]. Phytobiotic mixtures containing thymol, menthol, linalool, trans-anethole, 1,8-cineole, and related terpenoids were active against broiler-derived APEC and other *E. coli* strains, including colistin-resistant isolates [75]. Conversely, a phenolic-rich ethanolic extract of *Pereskia aculeata* showed measurable inhibition for only a limited subset of ESBL-associated *E. coli* isolates, with an MIC of 20 mg/mL where inhibition could be determined and no clear evidence that the ESBL phenotype altered susceptibility to the extract [76]. In vivo, caprylic-acid supplementation reduced cecal colonization by MDR *Salmonella* Heidelberg by approximately 3.6 log10 CFU/g [77], while a mixture of eugenol and trans-cinnamaldehyde reduced XDR *Campylobacter jejuni* loads in challenged broilers [23]. Marjoram essential oil also showed activity against MDR avian pathogens and, when combined with conventional antibiotics, improved outcomes in experimental bacterial leg-disorder models [78]. These findings are relevant to the control of resistant poultry-associated pathogens; however, reduced colonization or bacterial burden, including the observed reduction in MDR *Salmonella* Heidelberg, should not be interpreted as evidence of a reduced intestinal resistome. Demonstrating such an effect requires direct evaluation of resistance-related endpoints, including ARG abundance or resistome composition, rather than pathogen counts alone.

Broiler experiments also demonstrate why in vitro phytochemical efficacy cannot be assumed to translate into intestinal AMR mitigation. Coated trans-cinnamaldehyde showed marked anti-*Campylobacter* activity in vitro but failed to reduce *C. jejuni* colonization in the broiler cecum [79], and supplementation with several plant extracts and essential-oil constituents produced only limited or transient reductions in *Campylobacter* shedding in another feeding study [80]. Likewise, an essential-oil blend containing carvacrol, thymol, eucalyptol, and lemon components reduced *Salmonella* Heidelberg colonization in the crop but not cecal colonization or fecal shedding [81]. In contrast, trans-cinnamaldehyde and eugenol reduced *Salmonella* Enteritidis cecal populations by at least 3 log10 CFU/g in young broilers [82], and oregano essential oil reduced *S.* Enteritidis prevalence and cecal burden in market-age birds [83]. Methanolic extracts of ginger and onion [84] and of *Moringa oleifera* and *Thymus vulgaris* [85] have likewise reduced *E. coli* burdens in experimental broiler infection models. These contrasting results reinforce a central conclusion of the present review: direct broiler evidence spans a continuum from pathogen suppression to ARG and resistome modulation, but relatively few studies simultaneously evaluate resistance phenotype, ARG abundance or expression, MGEs, gene mobility, and community-level resistome dynamics. Representative studies and the level of AMR inference supported by each are summarized in Table 2.

## 8. Methodological Priorities and Future Research

Future studies should move beyond single-endpoint evaluation and adopt integrated experimental designs that connect phytochemical characterization with antimicrobial phenotype, microbiome structure, resistome composition, and resistance mobility. At a minimum, interventions should be chemically characterized, administered at a defined and biologically relevant dose, and evaluated longitudinally using age-matched controls, because bird age itself can exert a stronger effect on resistome structure than dietary treatment [14]. Relative ARG abundance alone is insufficient to distinguish genuine loss of resistance determinants from changes in community composition; therefore, absolute quantification approaches, including internal-standard or spike-in strategies, should increasingly complement conventional relative metagenomic analyses [86,87]. Such designs would make it possible to determine whether an apparent decrease in resistance reflects lower total ARG burden, redistribution among bacterial hosts, or simple compositional effects.

A second priority is to strengthen the linkage between resistance-gene detection, genetic context, expression, and phenotype. Shotgun metagenomics can define community-level resistance potential but does not by itself establish ARG expression, host identity, mobility, or phenotypic resistance [86]. Metatranscriptomics can add information on ARG transcripts, whereas long-read sequencing can improve reconstruction of plasmids and ARG-MGE-host associations, as recently demonstrated in broiler chickens [6,12,86]. Emerging approaches such as Hi-C sequencing may further resolve physical associations between ARGs, plasmids, and bacterial hosts in complex microbial communities [88]. Nevertheless, claims of active horizontal transfer should ultimately be supported by longitudinal tracking or functional conjugation assays, because MGE detection indicates transfer potential rather than transfer itself [15,86,89].

Finally, future broiler studies should extend sampling beyond the intestinal compartment. An optimal experimental framework would follow the same intervention from the phytochemically characterized feed additive through cecal and fecal microbiomes and litter or manure to selected environmental compartments, while simultaneously assessing resistant bacterial phenotypes, absolute ARG abundance, ARG expression, MGE linkage, and transfer potential. Repeated sampling across the production cycle would help separate treatment effects from age-related ecological succession, while post-excretion monitoring would establish whether intestinal changes persist after release into litter, soil, water, or crop-associated microbiomes [63,64,65,66,67,68,69,70,71]. Studies should also include repeated subinhibitory-exposure experiments where relevant, particularly for compounds intended for long-term dietary use, to determine whether AMR mitigation is accompanied by selection for phytochemical tolerance or antibiotic cross-resistance [56,57,58,59,60,61,62]. In this way, future research can progress from demonstrating that a phytogenic intervention changes the microbiome or resistome to establishing whether it produces a durable and biologically meaningful reduction in resistance risk across the poultry production continuum. The principal analytical approaches and the strength of inference supported by each are summarized in Table 3.

## 9. Translational and Regulatory Considerations for Implementation in Broiler Production

Translation of phytochemical-mediated AMR modulation from experimental models to commercial broiler production requires that biological activity be linked to actual exposure at the intestinal site of action. Nominal inclusion concentration in feed is an incomplete measure of biologically relevant exposure because volatility, feed processing, storage, carrier system, interactions with the dietary matrix, gastrointestinal absorption, and regional distribution can substantially modify the amount of active compound reaching bacterial communities [51,53,54,55,90,91]. This issue is especially relevant for essential-oil constituents. In an assessment of a partially microencapsulated thyme- and star-anise-based feed additive, measurable losses of thymol and trans-anethole occurred during pelleting and subsequent storage, illustrating that the composition of the feed ultimately consumed by the bird may differ from that of the original preparation [90]. Direct measurement of phytochemical distribution within broilers provides further evidence of this exposure gradient: following dietary supplementation, trans-cinnamaldehyde remained detectable throughout the gastrointestinal tract, including the ceca and colon, whereas caprylic acid was not detected in these distal compartments [91]. Consequently, future AMR-oriented studies should report not only the administered dietary dose but, where feasible, marker-compound concentrations after feed processing and within the intestinal compartment in which the resistance-modifying effect is expected to occur.

Product identity and compositional standardization are equally important for translation and regulatory interpretation. Within the European Union, feed additives are authorised according to Regulation (EC) No 1831/2003 and are classified according to their intended functions, while EFSA guidance requires appropriate characterisation of the additive, its active components, conditions of use, safety, and evidence of efficacy relevant to the claimed function [92,93,94]. For botanical preparations, this requirement is particularly consequential because a plant species name or generic designation such as “essential oil” does not define a reproducible intervention. An EFSA assessment of an oregano essential oil intended for poultry, for example, was unable to complete the risk assessment for the target species, consumer, and user because a substantial fraction of the preparation remained chemically uncharacterised [95]. Conversely, botanical compounds may be authorised as sensory additives on the basis of their flavouring function without this implying efficacy as resistance-modifying agents [92,95]. Thus, regulatory acceptance for one intended purpose should not be interpreted as evidence of AMR mitigation. If resistance modification, ARG reduction, inhibition of horizontal transfer, or resistome modulation is proposed as a functional benefit, the supporting evidence should include AMR-specific endpoints of the type distinguished throughout this review rather than relying solely on growth performance, palatability, microbiota composition, or general antibacterial activity.

Safety evaluation must similarly move beyond the assumption that botanical origin implies intrinsically low risk. Feed-additive assessment encompasses safety for the target animal, the consumer, the user handling the product, and the environment, and conclusions are dependent on both composition and conditions of use [90,92,93,94,95,96]. In a recent EFSA assessment of 41 herbal flavouring compounds, tolerance studies in chickens for fattening and other target species supported safety at the proposed inclusion levels, with no adverse effects observed for the tested compounds at substantially higher experimental exposure; nevertheless, those conclusions applied to chemically defined substances and specified use conditions [96]. Pharmacokinetic and residue considerations may also become relevant as dietary exposure increases. In broilers receiving graded concentrations of thyme essential oil, thymol deposition was lowest in breast muscle but increased in plasma and kidney and became detectable in liver and muscle at the highest dietary concentration tested [97]. Dose selection should therefore balance sufficient intestinal exposure with target-species tolerability and consumer safety. From an AMR perspective, this balance has an additional dimension: concentrations should also be evaluated for their potential to generate prolonged subinhibitory exposure, which, as discussed in Section 5, may favour bacterial adaptation under some experimental conditions [56,57,58,59,60,61,62].

Finally, successful implementation requires evidence that efficacy is reproducible under commercial production conditions rather than confined to highly controlled challenge experiments. Four large commercial-farm trials involving an encapsulated cinnamaldehyde product demonstrated that phytogenic supplementation can be incorporated into pelleted diets and evaluated under field conditions involving different broiler flocks and production stressors [98]. Earlier multi-location trials of a thymol-, eugenol-, and piperine-containing feed additive likewise showed that responses can vary among production settings and that dietary dose influences the consistency of biological effects [99]. More recently, a large broiler experiment evaluating carvacrol- and thymol-based feed additives found little adverse impact on carcass yield or most breast-meat quality traits, providing useful information on compatibility with production endpoints [100]. However, commercial-scale studies of this type have generally focused on growth, intestinal health, pathogen control, or product quality rather than resistance phenotype, ARG burden, MGE mobility, or resistome structure. The next translational step should therefore be multi-flock and preferably multi-site trials using chemically standardised formulations at commercially realistic doses while incorporating longitudinal AMR-specific endpoints, exposure measurements, safety outcomes, and production performance. Commercial adoption of phytogenic interventions as tools for AMR mitigation should ultimately depend not on their designation as “natural antibiotic alternatives”, but on reproducible evidence that a defined product produces a meaningful reduction in resistance risk under realistic poultry-production conditions.

## 10. Conclusions

Plant-derived bioactive compounds represent promising tools for antimicrobial-resistance management in broiler production, but the available evidence clearly indicates that antimicrobial activity against resistant bacteria should not be equated with AMR mitigation. Stronger evidence requires demonstration of altered antimicrobial susceptibility, interference with defined resistance mechanisms, modulation of ARGs and MGEs, inhibition of horizontal gene transfer, or reproducible changes in the community-level resistome. Broiler-specific studies provide encouraging examples across these endpoints, yet the responses remain heterogeneous and are strongly influenced by phytochemical composition, dose, formulation, intestinal exposure, bird age, microbial ecology, and the analytical methods used. Importantly, inconsistent in vivo efficacy and the potential for adaptation following repeated subinhibitory exposure further argue against assuming that plant-derived interventions are intrinsically resistance-suppressive.

The future value of phytogenic strategies will therefore depend on moving from proof-of-principle antimicrobial effects toward chemically standardized, biologically relevant interventions that demonstrate durable reductions in resistance phenotype, ARG burden or mobility, and dissemination potential under realistic broiler-production conditions. Integrated longitudinal studies combining phenotypic susceptibility testing with quantitative resistome analysis, ARG–host–MGE resolution, functional transfer assays, and follow-up across litter and environmental compartments will be essential to determine whether intestinal effects translate into meaningful One Health benefits. Such evidence would provide a stronger basis for incorporating plant-derived bioactive compounds into antimicrobial-stewardship programmes as complementary tools for reducing resistance risk rather than simply as natural substitutes for conventional antibiotics.

## Figures and Tables

**Figure 1:**
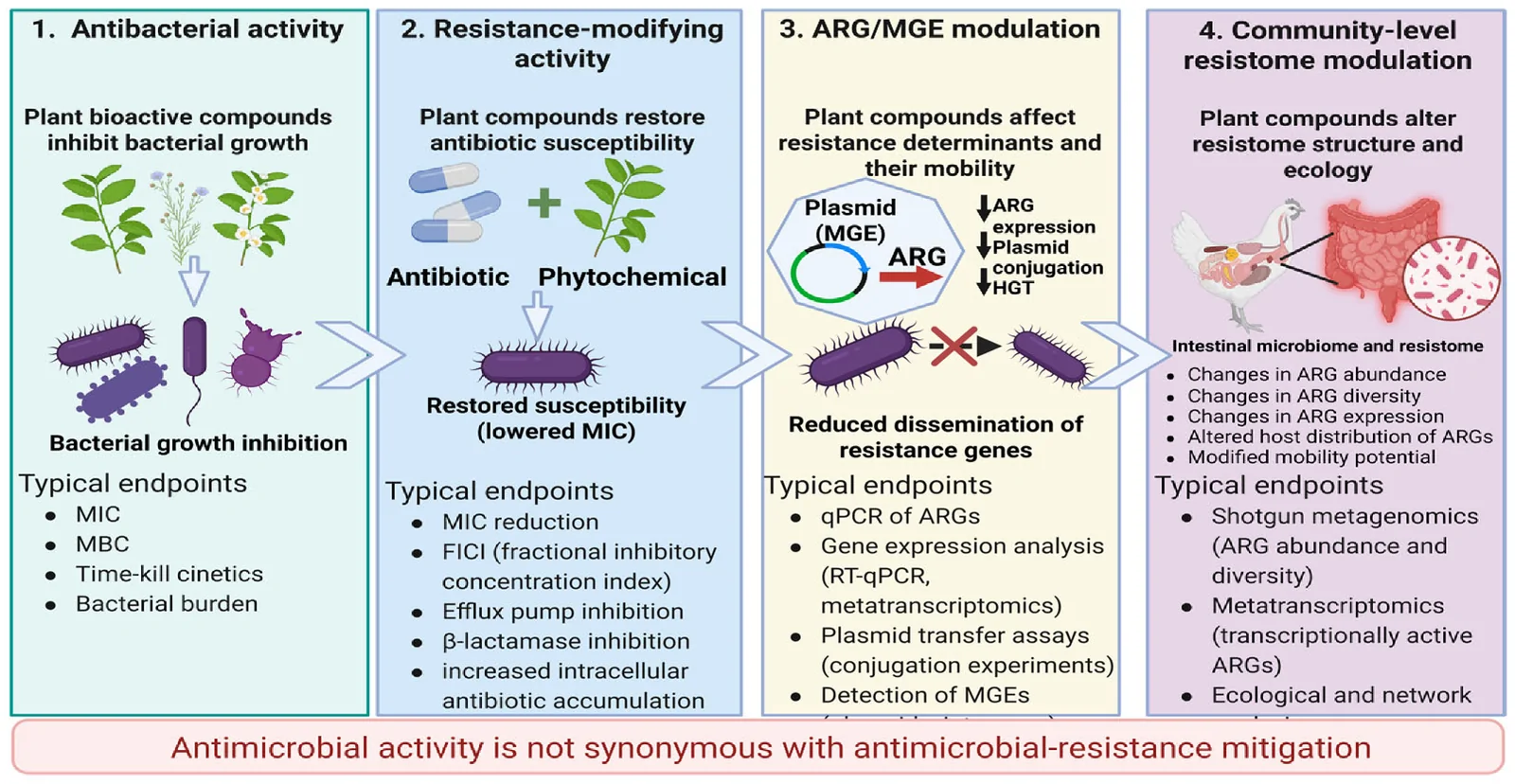
Evidence continuum from antibacterial activity to antimicrobial-resistance mitigation by plant-derived bioactive compounds. (Created in BioRender. Nadas, N. (2026) https://BioRender.com/kkcycqv (accessed on 12 September 2026)).

**Figure 2:**
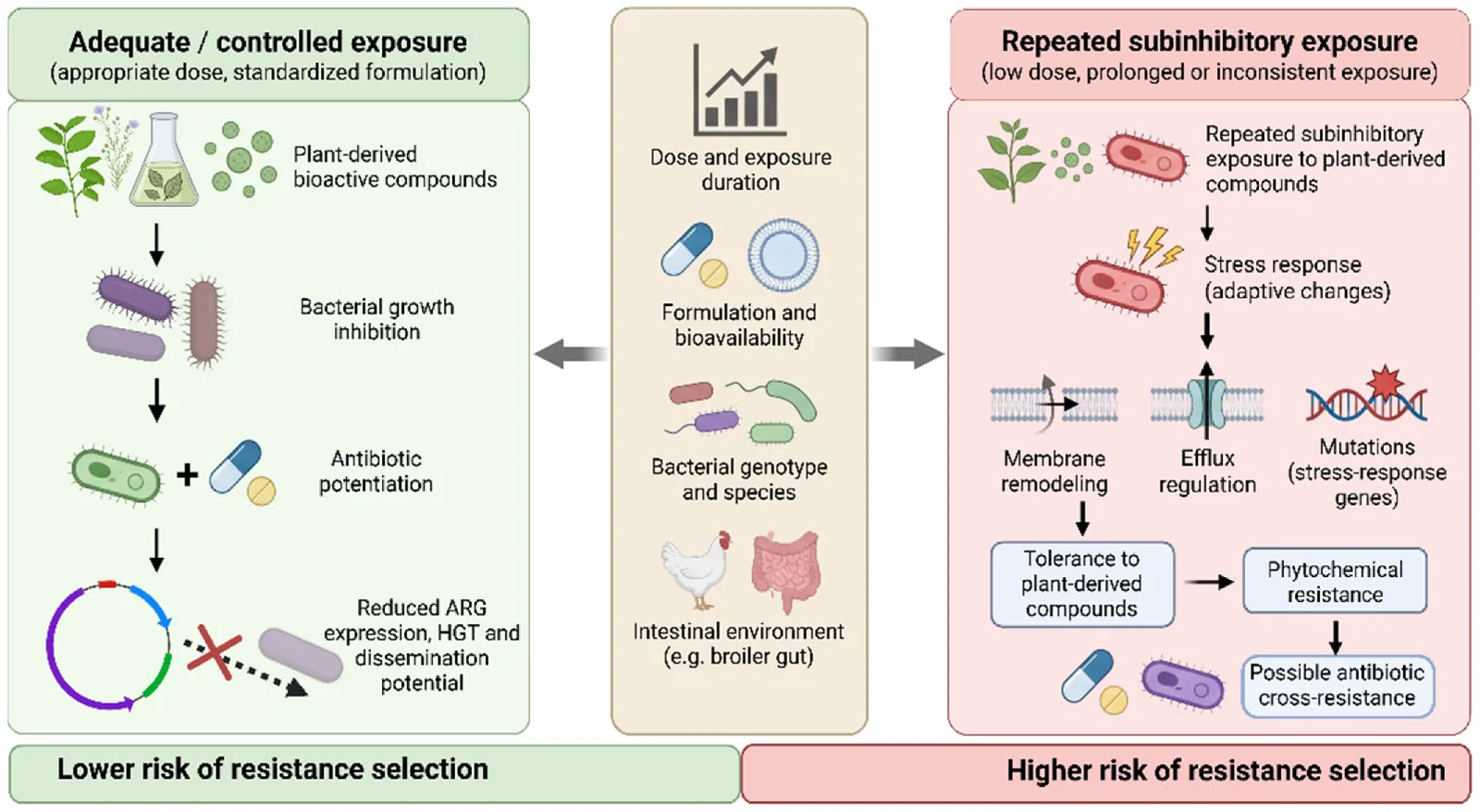
Exposure-dependent outcomes of plant-derived antimicrobials. Controlled exposure may favor antibacterial activity and antibiotic potentiation, whereas repeated subinhibitory exposure may promote adaptive responses, tolerance, phytochemical resistance, and potential antibiotic cross-resistance. These outcomes are influenced by dose, exposure duration, formulation, bacterial genotype, and the intestinal environment. ARG, antimicrobial resistance gene; HGT, horizontal gene transfer. (Created in BioRender. Nadas, N. (2026) https://BioRender.com/udhgxla). https://app.biorender.com/illustrations/6aa66d00f307511ee27a8cad?slideId=094cf38e-9876-4293-b99a-9856d338cd39 accessed on 15 September 2026.

**Figure 3:**
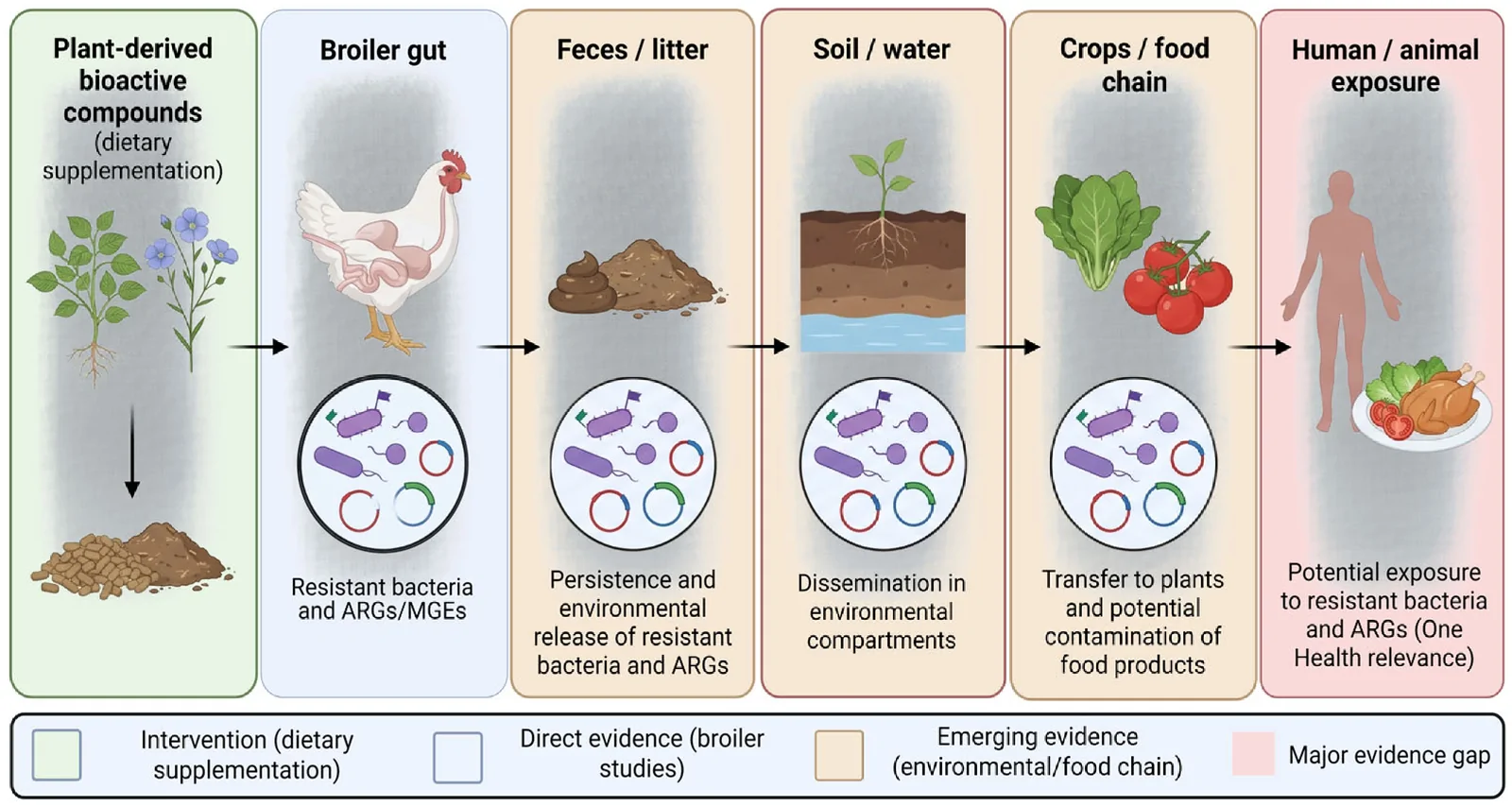
One Health continuum linking the broiler gut resistome with feces and litter, environmental compartments, crops and the food chain, and potential human or animal exposure. Green identifies the plant-derived dietary intervention. Blue indicates direct evidence from broiler studies, beige indicates emerging evidence from environmental and food-chain studies, and pink indicates major evidence gaps. (Created in BioRender. Nadas, N. (2026) https://BioRender.com/kq0oi07 accessed on 12 September 2026).

**Table 1:** Major classes of plant-derived bioactive compounds with AMR-relevant activities and their level of poultry evidence.

Phytochemical Class	Representative Compounds and Plant Sources	Key Structural/Physicochemical Features	AMR-Relevant Activity and Representative Mechanism	Evidence Level	Standardization/Formulation Considerations	References
Phenolic monoterpenes	Carvacrol, thymol; *Origanum*, *Thymus* spp.	Hydrophobic aromatic scaffold with a phenolic hydroxyl group	Antibacterial activity and antibiotic potentiation; membrane perturbation and EtBr accumulation consistent with altered efflux-related activity; inhibition of plasmid conjugation in experimental models	Direct broiler and poultry-associated	Strongly dependent on essential-oil chemotype, constituent ratios, volatility, and encapsulation	[15,28,37,44,51,52,53,54,55]
Phenylpropanoids	trans-cinnamaldehyde, eugenol; *Cinnamomum* spp., *Syzygium aromaticum*	Aromatic aldehyde or phenolic structures; relatively lipophilic	Antibacterial activity, antibiotic potentiation, and HGT inhibition; membrane effects, altered antimicrobial susceptibility, and interference with conjugation-associated processes	Direct broiler and poultry-associated	Stability and intestinal exposure are influenced by formulation and carrier system	[15,23,25,26,27,51,54,55]
Flavonoids	Scutellarein, prenylated flavonoids, EGCG; diverse medicinal and food plants	Activity influenced by hydroxylation, prenylation, methoxylation, polarity, and lipophilicity	Efflux inhibition, anti-conjugation activity, and antibiotic potentiation; NorA inhibition, envelope stress, metal-homeostasis disruption, and cell-wall effects	Mechanistic/non-poultry	Extraction solvent and substitution pattern strongly influence recovered compounds and biological activity	[38,45,46,48,49,50]
Alkaloids	Berberine; *Berberis* spp.	Cationic isoquinoline structure; intracellular accumulation limited by efflux	Antibacterial activity and potentiation in the presence of efflux inhibitors; increased intracellular accumulation following efflux inhibition	Mechanistic/non-poultry	Activity depends on co-occurring resistance-modifying constituents and overall extract composition	[47]
Isothiocyanates	2-Phenylethyl isothiocyanate; Brassicaceae	Electrophilic –N=C=S functional group	Antibiotic potentiation through interference with bacterial cellular targets and resistance-associated processes	Mechanistic/non-poultry	Concentration and extraction procedure influence compound availability	[29,50]
Quinones	Primin; Primulaceae	Redox-active benzoquinone scaffold	β-Lactam potentiation through direct inhibition of NDM-type metallo-β-lactamase activity	Mechanistic/non-poultry	Use of a defined purified compound provides greater chemical reproducibility than crude extracts	[31]
Saponins	*Pulsatilla* saponins; *Pulsatilla* spp.	Amphiphilic glycosides	ARG and resistome modulation, predominantly through microbiome- and ecology-mediated effects	Direct broiler	Botanical source, extraction procedure, purity, and administered dose require standardization	[34,50]
Polyphenol-rich preparations	Cranberry, blueberry, and blackberry pomaces; berry-processing by-products	Complex mixtures of phenolic acids, flavonoids, anthocyanins, and other polyphenols	Community-level resistome modulation associated with microbiome restructuring and altered ARG distribution	Direct broiler	Composition varies with cultivar, processing conditions, extraction procedure, and batch	[39,40,41,45,49,50]

Abbreviations: AMR, antimicrobial resistance; ARG, antimicrobial resistance gene; EGCG, epigallocatechin gallate; HGT, horizontal gene transfer; NDM, New Delhi metallo-β-lactamase.

**Table 2:** Representative evidence for phytochemical effects on antimicrobial resistance-related endpoints, grouped by evidence category and experimental model.

Intervention	Experimental System	AMR-Related Endpoint	Main Finding	Level of Inference/Caution	Refs.
A. Antibacterial activity against resistant pathogens and related pathogen-load outcomes					
Multicomponent phytobiotic mixtures	Broiler-derived APEC/*E. coli* isolates	MIC; growth inhibition	Antibacterial activity against APEC, including colistin-resistant isolates.	Poultry isolate; antibacterial effect only.	[75]
Pereskia aculeata extract	ESBL-associated *E. coli* from chicks/broilers	MIC/MBC; ESBL status	Limited inhibition; susceptibility not clearly associated with ESBL status.	Poultry isolate; no demonstrated resistance reversal.	[76]
Caprylic acid	MDR *S.* Heidelberg-challenged broilers	Cecal bacterial load	Approximately 3.6-log10 CFU/g reduction in cecal pathogen counts.	In vivo broiler; pathogen reduction, not resistome change.	[77]
Eugenol + trans-cinnamaldehyde	XDR *C. jejuni*-challenged broilers	Cecal load; virulence transcripts	Reduced cecal burden and expression of selected virulence genes.	In vivo broiler; not an ARG/resistome endpoint.	[23]
Marjoram essential oil ± antibiotics	MDR avian-pathogen experimental models	Pathogen burden; treatment response	Improved antimicrobial/treatment outcome in combination experiments.	Poultry-related treatment efficacy; mechanism not established.	[78]
Commercial phytogenic additive	Broilers given carvacrol/cinnamaldehyde/eugenol preparation	Prevalence of ESBL-producing Enterobacteriaceae	Lower prevalence of ESBL-producing Enterobacteriaceae.	In vivo broiler; bacterial resistance phenotype prevalence, not proof of HGT suppression.	[33]
Coated trans-cinnamaldehyde	*C. jejuni*-challenged broilers	Cecal colonization	Pronounced in vitro effect but no significant in vivo reduction.	Negative translation; no ARG/resistome measurement.	[79]
Plant extracts/essential-oil constituents	*C. jejuni*-colonized broilers	Fecal/cecal pathogen load	Limited or transient reduction in bacterial shedding/burden.	In vivo antibacterial outcome; resistance status not established.	[80]
trans-cinnamaldehyde + eugenol	*S.* Enteritidis-challenged broilers	Cecal bacterial load	At least 3-log10 CFU/g reduction in cecal population.	In vivo antibacterial outcome; resistance status not established.	[82]
Oregano essential oil	*S.* Enteritidis-challenged market-age broilers	Prevalence; cecal load	Reduced prevalence and approximately 1-log10 cecal reduction.	In vivo antibacterial outcome; resistance status not established.	[83]
Essential-oil blend (carvacrol/thymol/eucalyptol/lemon)	*S.* Heidelberg-challenged broilers	Crop/cecal colonization; shedding	Reduced crop colonization, but not cecal colonization or fecal shedding.	Site-specific antibacterial outcome; no resistome endpoint.	[81]
B. Altered antimicrobial susceptibility and antibiotic potentiation					
Lavender, cinnamon bark or clove oils + enrofloxacin	MDR *E. coli* isolates from day-old broilers; in vitro	Checkerboard FICI; time–kill	Reported synergy/additivity; frequency varied with the oil and combinations.	Poultry isolate; combination phenotype, not mechanism.	[25]
Sub-MIC trans-cinnamaldehyde, thymol, carvacrol, eugenol or β-resorcylic acid	MDR *S.* Typhimurium DT104; in vitro	Antibiotic sensitivity; growth-based testing	Enhanced activity of selected antibiotics under the test conditions.	In vitro growth/susceptibility effect; not checkerboard-defined synergy.	[26]
Thymol, carvacrol or cinnamaldehyde + antibiotics	Resistant *Salmonella*, *E. coli* and *S. aureus* strains; in vitro	MIC; checkerboard FICI	Selected combinations reduced antibiotic MICs.	Non-broiler/mixed-isolate phenotypic potentiation.	[27]
Watercress extract or 2-phenylethyl isothiocyanate + antibiotics	ESBL *E. coli* of clinical/animal origin; in vitro	Disk diffusion; MIC-based assays	Enhanced antibacterial effect in selected combinations.	In vitro potentiation; does not prove β-lactamase inhibition.	[29]
Encapsulated cinnamaldehyde ± citral	In vivo broilers; fecal *E. coli* isolates	AST; WGS; genotype (see D)	Lower resistance to selected antibiotics in some comparisons.	In vivo broiler phenotype; endpoint- and drug-specific.	[72]
Organic acids + cinnamaldehyde	MDR APEC-challenged broilers	Resistant *E. coli* counts; ceftriaxone MIC	No enrichment of resistant *E. coli*; lower ceftriaxone MIC than antibiotic group.	In vivo broiler; mixed formulation; not specific mechanistic proof.	[73]
C. Resistance-mechanism modulation					
Thymol or carvacrol	Foodborne bacterial pathogens; in vitro	EtBr accumulation; tetracycline MIC	EtBr accumulation increased; tetracycline MIC declined two- to eight-fold.	Consistent with altered efflux; membrane permeability is a confounder.	[28]
Carvacrol, thymol or eugenol	*S.* Typhimurium; in vitro	EtBr accumulation; nalidixic-acid susceptibility; biofilm	Altered dye accumulation and susceptibility, with membrane-associated effects.	Efflux-associated phenotype; pump-specific inhibition unconfirmed.	[32]
Selected medicinal-plant extracts	NDM-1 enzyme and NDM-1-producing *E. coli*; in vitro	Purified-enzyme assay; checkerboard	NDM-1 inhibition measured biochemically; antibiotic-combination effects observed separately.	Direct enzyme evidence versus bacterial phenotypic potentiation.	[30]
Primin + meropenem	NDM-5 enzyme, *E. coli* and mouse infection model	Biochemical inhibition; MIC/FICI/time–kill; murine efficacy	NDM-5 inhibited in biochemical tests; meropenem activity enhanced, including in mice.	Mechanistic plus non-poultry in vivo proof of principle; no broiler efficacy established.	[31]
D. ARG or mobile genetic element (MGE) changes					
Encapsulated cinnamaldehyde ± citral	In vivo broilers; fecal *E. coli*	WGS; selected ARGs/plasmid markers	Lower prevalence of some ARGs/plasmid markers in investigated isolates.	Broiler isolate genotype; not community-wide resistome loss.	[72]
Commercial phytogenic additive	In vivo broilers; gut microbial community	Metatranscriptomics; ARG transcripts	Changed/reduced ARG transcript abundance in comparisons reported.	Community transcript endpoint; functional resistance not directly established.	[12]
Neolamarckia cadamba leaf extract	In vivo broilers; cecal/fecal samples	ARG/MGE abundance; potential host associations	Changes in selected ARG/MGE profiles and inferred bacterial associations.	Broiler ARG/MGE endpoint; host assignments may be method-limited.	[13]
Allicin + cinnamon oil	MDR broiler-derived *E. coli*; in vitro	intI1, fimH, luxS expression (qRT-PCR)	Reduced intI1 and virulence-associated fimH/luxS transcripts.	Poultry isolate; intI1 is an integron marker, not an ARG; transcripts ≠ functional resistance.	[74]
E. Horizontal gene transfer (HGT)/plasmid-conjugation inhibition					
trans-cinnamaldehyde, carvacrol, β-resorcylic acid or caprylic acid	Defined donor–recipient assays in broth/manure/water; broiler cecal contents ex vivo	Plasmid-conjugation frequency	Reduced transfer of *bla*_TEM_-associated plasmid in tested systems.	Poultry-associated in vitro/ex vivo; no direct within-bird HGT measurement.	[15]
Commercial carvacrol/cinnamaldehyde/eugenol phytogenics	Broilers fed supplements; conjugation tested ex vivo	ESBL plasmid transfer assays	Conjugation effects were inconsistent despite lower ESBL-carrier prevalence.	Broiler feeding + ex vivo functional endpoint; not consistent HGT inhibition.	[33]
Linalool (Thymus vulgaris chemistry)	*E. coli* pKM101 conjugation; in vitro	Plasmid transfer frequency	Approximately 84% lower transfer under the reported experimental conditions.	Non-poultry mechanistic proof of principle.	[35]
Nanocarriers with sage or olibanum essential oils	Experimental food/chicken-derived matrices; ex vivo	*bla*_TEM_-associated conjugation	Reduced conjugative transfer in tested matrices.	Poultry-associated matrix; not an in vivo broiler result.	[36]
Carvacrol or thymol	IncX3 blaNDM plasmid in bacterial systems; non-poultry animal gut model	Inter-/intrageneric conjugation; bacterial physiology	Reduced plasmid transfer with associated metabolic/membrane changes.	Mechanistic plus non-poultry animal evidence; possible indirect effects.	[37]
Selected flavonoids, including scutellarein	Bacterial conjugation assays and murine intestinal models	Plasmid transfer; iron/zinc stress pathways	Conjugation reduction associated with altered metal homeostasis and cellular stress.	Non-poultry in vitro/murine proof of principle; not broiler validation.	[38]
F. Community-level resistome modulation					
Pulsatilla saponins + herbal formulation	In vivo broilers	ARG categories; resistome abundance/diversity	Reduced selected multidrug resistance-related ARG categories in reported comparisons.	Direct broiler resistome outcome; comparison-dependent.	[34]
Berry pomace/polyphenol-rich preparations	In vivo broilers; cecal metagenomics	Resistome composition; ARG categories	Resistome shifts with gene-specific changes, not uniform ARG suppression.	Direct broiler resistome; ecological composition may contribute.	[39,40,41]
Essential-oil blend	In vivo broilers; shotgun metagenomics	Resistome α-/β-diversity; ARG repertoire	No significant treatment effect on diversity; bird age was more influential.	Direct neutral/negative resistome evidence.	[14]

Notes. Categories describe measured endpoints, not interchangeable levels of AMR mitigation. Some references appear in more than one category when distinct endpoints were investigated. Studies reporting only bacterial-colonization outcomes without a confirmed resistant phenotype are retained in category A as contextual antibacterial evidence and are explicitly identified. EtBr, ethidium bromide; ESBL, extended-spectrum β-lactamase; FICI, fractional inhibitory concentration index; HGT, horizontal gene transfer; MGE, mobile genetic element.

**Table 3:** Analytical approaches for evaluating antimicrobial-resistance modulation and the strength of inference supported by each method.

Analytical Approach	What It Can Demonstrate	What It Cannot Demonstrate Alone	Main Limitation/Interpretative Caution	References
Culture-based antimicrobial susceptibility testing (MIC/MBC, disk diffusion)	Phenotypic susceptibility or resistance; changes in MIC/MBC following phytochemical exposure	ARG identity, gene expression, mobility, or community-level resistome effects	Limited to cultivable organisms and tested antimicrobial–organism combinations	[17,20,25,26,27,28]
Checkerboard and time–kill assays	Synergy, additivity, antagonism, and antibiotic potentiation	Molecular mechanism responsible for synergy or resistance reversal	FICI-based synergy does not establish efflux inhibition, enzyme inhibition, or in vivo relevance	[25,26,27,29,30,31]
qPCR/digital PCR	Targeted relative or absolute quantification of selected ARGs or MGEs	Resistome-wide diversity, host identity, genomic location, or transfer activity	Requires predefined targets; relative abundance may be confounded by changes in microbial community composition	[13,66,87]
Whole-genome sequencing of cultured isolates	Resistance genotype, mutations, plasmid content, and genotype–phenotype associations in individual isolates	Community-wide resistome structure or transfer dynamics within the complete microbiota	Restricted to recovered isolates; detection of a resistance determinant does not establish expression or mobility	[61,62,72]
Shotgun metagenomics	Resistome composition, ARG richness/diversity, taxonomic context, and community-level changes	Whether detected ARGs are expressed or actively transferred	Short reads may limit ARG–host and ARG–MGE linkage; abundance is often reported relatively unless an absolute-quantification strategy is incorporated	[14,39,40,41,42,43,86]
Metatranscriptomics	Transcriptionally active resistance determinants and functional resistome responses	Genomic location, plasmid association, or actual HGT events	RNA expression is temporally dynamic and does not necessarily predict phenotypic resistance	[12,86]
Long-read/plasmid-resolved sequencing	Improved reconstruction of plasmids, ARG–MGE associations, genomic context, and potential bacterial hosts	Whether identified MGEs are actively transferring under experimental conditions	Higher cost, lower throughput in some designs, and greater bioinformatic complexity	[6,86]
Hi-C metagenomics	Proximity-based linkage of ARGs, plasmids, and bacterial hosts within complex communities	Direct proof that conjugation or HGT is occurring	Contact-based associations require careful interpretation and independent validation	[88]
Functional conjugation/plasmid-transfer assays	Direct measurement of horizontal transfer frequency between defined donor and recipient populations	Community-wide resistome dynamics or transfer across the complete microbiota	Simplified donor–recipient systems may not reproduce intestinal ecological complexity	[15,33,35,36,37,38,89]
Longitudinal microbiome–resistome profiling	Temporal treatment effects, persistence, age-related trajectories, and post-intervention changes	Mechanistic causality unless combined with functional or molecular assays	Requires repeated sampling, appropriate controls, and separation of treatment effects from age-related ecological succession	[6,14,63,64]
Integrated gut-litter-environment sampling	Persistence and dissemination of resistant bacteria, ARGs, and MGEs beyond the intestinal compartment	Mobility, expression, or clinical relevance of every detected resistance determinant	Environmental dilution, multiple sources, temporal variability, and difficulty establishing direct transmission pathways	[63,64,65,66,67,68,69,70,71]

Abbreviations: ARG, antimicrobial resistance gene; FICI, fractional inhibitory concentration index; HGT, horizontal gene transfer; MBC, minimum bactericidal concentration; MGE, mobile genetic element; MIC, minimum inhibitory concentration; PCR, polymerase chain reaction; qPCR, quantitative polymerase chain reaction.

## Data Availability

No new data were created or analyzed in this study. Data sharing is not applicable to this article.

## Supplementary Materials

The following supporting information can be downloaded at https://www.mdpi.com/article/10.3390/plants15213285/s1. Table S1: Database-specific literature search strategies, search fields, publication-date restrictions, and retrieval counts.
